# (Acetyl­acetone isonicotinoylhydrazonato-κ^3^ *O*,*N*′,*O*′)dioxidovanadate(V) monohydrate

**DOI:** 10.1107/S1600536810028886

**Published:** 2010-07-24

**Authors:** Hon Wee Wong, Kong Mun Lo, Seik Weng Ng

**Affiliations:** a Department of Chemistry, University of Malaya, 50603 Kuala Lumpur, Malaysia

## Abstract

The hydrazone anion in the title compound, [V(C_11_H_12_N_3_O_2_)O_2_]·H_2_O, is zwitterionic as its pyridyl N atom is protonated; the O, N and O′ atoms span the axial–equatorial–axial positions of the trigonal-bipyramidal coord­in­ation polyhedron of the metal atom. All non-H atoms lie on a crystallographic mirror plane apart from the oxide ligands, which are related by mirror symmetry. The pyridinium N atom acts as a hydrogen-bond donor to the solvent water mol­ecule, which is in turn a hydrogen-bond donor to the both oxide ligands. These hydrogen-bonding inter­actions give rise to a three-dimensional network motif.

## Related literature

For related vanadium(V) structures, see: Shao *et al.* (1988 ▶). The reaction of oxidovanadium(IV) bis­(acetyl­acetonate), VO(acac)_2_, with aroylhydrazines in methanol yields Schiff-base complexes having the dinuclear [V(=O)(*μ*-OMe)_2_V(=O)]^4+^ core, see: Sarkari & Pal (2009 ▶).
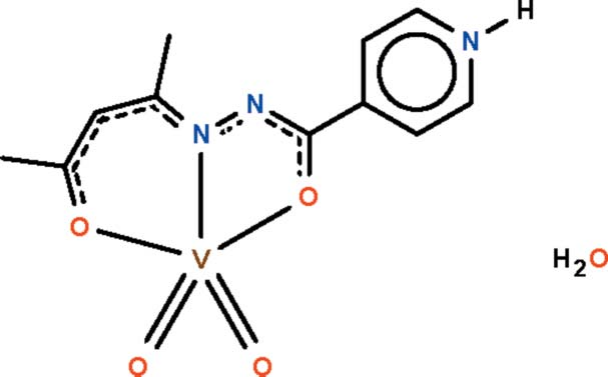


## Experimental

### 

#### Crystal data

[V(C_11_H_12_N_3_O_2_)O_2_]·H_2_O
*M* *_r_* = 319.19
Orthorhombic, 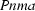

*a* = 13.9848 (10) Å
*b* = 6.6630 (4) Å
*c* = 13.8904 (10) Å
*V* = 1294.32 (15) Å^3^
*Z* = 4
Mo *K*α radiation
μ = 0.79 mm^−1^
*T* = 100 K
0.35 × 0.20 × 0.20 mm

#### Data collection

Bruker SMART APEX diffractometer
Absorption correction: multi-scan (*SADABS*; Sheldrick, 1996 ▶) *T*_min_ = 0.770, *T*_max_ = 0.858
11995 measured reflections
1610 independent reflections
1416 reflections with *I* > 2σ(*I*)
*R*_int_ = 0.033

#### Refinement

*R*[*F* ^2^ > 2σ(*F* ^2^)] = 0.039
*wR*(*F* ^2^) = 0.121
*S* = 1.11
1610 reflections
125 parameters
2 restraints
H atoms treated by a mixture of independent and constrained refinement
Δρ_max_ = 0.75 e Å^−3^
Δρ_min_ = −0.72 e Å^−3^

### 

Data collection: *APEX2* (Bruker, 2009 ▶); cell refinement: *SAINT* (Bruker, 2009 ▶); data reduction: *SAINT*; program(s) used to solve structure: *SHELXS97* (Sheldrick, 2008 ▶); program(s) used to refine structure: *SHELXL97* (Sheldrick, 2008 ▶); molecular graphics: *X-SEED* (Barbour, 2001 ▶); software used to prepare material for publication: *publCIF* (Westrip, 2010 ▶).

## Supplementary Material

Crystal structure: contains datablocks global, I. DOI: 10.1107/S1600536810028886/nk2049sup1.cif

Structure factors: contains datablocks I. DOI: 10.1107/S1600536810028886/nk2049Isup2.hkl

Additional supplementary materials:  crystallographic information; 3D view; checkCIF report

## Figures and Tables

**Table 1:** Hydrogen-bond geometry (Å, °)

*D*—H⋯*A*	*D*—H	H⋯*A*	*D*⋯*A*	*D*—H⋯*A*
O1*W*—H1w⋯O1	0.84 (1)	1.91 (1)	2.732 (2)	168 (3)
N3—H3⋯O1w^i^	0.86 (1)	1.87 (3)	2.683 (4)	158 (6)

Symmetry code: (i) 
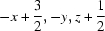
.

**Fig. 1.**
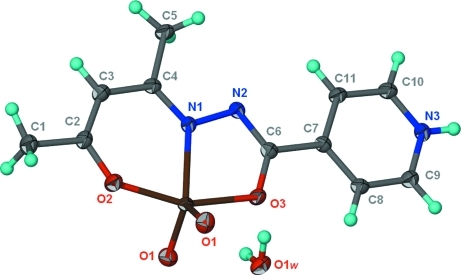
Thermal ellipsoid plot (Barbour, 2001) of VO2(C11H12N3O2).H2O at the 70% probability level; hydrogen atoms are drawn as spheres of arbitrary radius. Symmetry transformation: i = x, 1/2 – y, z.

## supplementary crystallographic information

### Comment

The reaction of oxovanadium(IV) bis(acetylacetonate), VO(acac)_2_, with aroylhydrazines in acetonitrile yields vanadium(V) compounds of the formulation V_2_O_3_*L*_2_ (where *L* represents the doubly-deprotonated Schiff base). In methanol, the reaction yields Schiff-base complexes having the dinuclear [V(=O)(*µ*-OMe)_2_V(=O)]^4+^ core (Sarkari & Pal, 2009). In the present study, the reaction with isonicotinic acid hydrazide yields the expected vanadium(V) complex of the mono-deprotonated Schiff base as a negatively-charged zwitterion as the pyridyl N-atom is protonated (Scheme I). The metal atom shows trigonal bipyramidal coordination, with the *O*,*N*,*O*'-atoms of the Schiff base spanning the axial sites (Fig. 1).

All non-hydrogen atoms lie on a crystallographic mirror plane other than the oxo ligands, which are related by mirror symmetry. The pyridinium N atom acts as a hydrogen-bond donor to the solvate water molecule, which is in turn a hydrogen bond donor to the both oxo ligands. Hydrogen bonding gives rise to a three-dimensional network motif.

### Experimental

Bis(acetylacetonato)oxovanadium(IV) (0.13 g, 0.5 mmol) and isonicotinic acid hydrazide (0.07 g, 0.75 mmol) heated in methanol (50 ml) for one hour. The brown solution was filtered; slow evaporation of the filtrate afforded brown crystals.

### Refinement

Carbon-bound H-atoms were placed in calculated positions (C—H 0.95 to 0.98 Å) and were included in the refinement in the riding model approximation, with *U*(H) set to 1.2 to 1.5*U*(C). The methyl carbons lies on a mirror plane, so that one of the H atoms lies on the plane whereas the other lies on a general position.

The amino and water H-atoms were located in a difference Fourier map, and were refined with distance restraints of N–H 0.86±0.01 and O–H 0.84±0.01 Å; their temperature factors were freely refined.

### Crystal data

**Table:**

[V(C_11_H_12_N_3_O_2_)O_2_]·H_2_O	*F*(000) = 656
*M**_r_* = 319.19	*D*_x_ = 1.638 Mg m^−^^3^
Orthorhombic, *P**n**m**a*	Mo *K*α radiation, λ = 0.71073 Å
Hall symbol: -P 2ac 2n	Cell parameters from 3731 reflections
*a* = 13.9848 (10) Å	θ = 2.9–27.6°
*b* = 6.6630 (4) Å	µ = 0.79 mm^−^^1^
*c* = 13.8904 (10) Å	*T* = 100 K
*V* = 1294.32 (15) Å^3^	Prism, brown
*Z* = 4	0.35 × 0.20 × 0.20 mm

### Data collection

**Table:**

Bruker SMART APEX diffractometer	1610 independent reflections
Radiation source: fine-focus sealed tube	1416 reflections with *I* > 2σ(*I*)
graphite	*R*_int_ = 0.033
ω scans	θ_max_ = 27.5°, θ_min_ = 2.1°
Absorption correction: multi-scan (*SADABS*; Sheldrick, 1996)	*h* = −18→18
*T*_min_ = 0.770, *T*_max_ = 0.858	*k* = −8→8
11995 measured reflections	*l* = −17→18

### Refinement

**Table:**

Refinement on *F*^2^	Primary atom site location: structure-invariant direct methods
Least-squares matrix: full	Secondary atom site location: difference Fourier map
*R*[*F*^2^ > 2σ(*F*^2^)] = 0.039	Hydrogen site location: inferred from neighbouring sites
*wR*(*F*^2^) = 0.121	H atoms treated by a mixture of independent and constrained refinement
*S* = 1.11	*w* = 1/[σ^2^(*F*_o_^2^) + (0.0616*P*)^2^ + 2.2562*P*] where *P* = (*F*_o_^2^ + 2*F*_c_^2^)/3
1610 reflections	(Δ/σ)_max_ = 0.001
125 parameters	Δρ_max_ = 0.75 e Å^−^^3^
2 restraints	Δρ_min_ = −0.72 e Å^−^^3^

### Fractional atomic coordinates and isotropic or equivalent isotropic displacement parameters (Å^2^)

**Table:**

	*x*	*y*	*z*	*U*_iso_*/*U*_eq_	Occ. (<1)
V1	0.41194 (4)	0.2500	0.31526 (4)	0.01205 (19)	
O1	0.41890 (11)	0.0504 (3)	0.24747 (12)	0.0191 (4)	
O2	0.27403 (16)	0.2500	0.32874 (16)	0.0204 (5)	
O3	0.54634 (16)	0.2500	0.35590 (16)	0.0189 (5)	
O1W	0.47876 (17)	−0.2500	0.12798 (18)	0.0194 (5)	
H1W	0.467 (2)	−0.148 (3)	0.161 (2)	0.040 (10)*	
N1	0.40746 (18)	0.2500	0.46744 (19)	0.0138 (5)	
N2	0.49679 (19)	0.2500	0.51358 (19)	0.0139 (5)	
N3	0.84943 (19)	0.2500	0.5420 (2)	0.0155 (5)	
H3	0.9099 (11)	0.2500	0.554 (4)	0.059 (18)*	
C1	0.1102 (2)	0.2500	0.3685 (3)	0.0218 (7)	
H1A	0.1065	0.2500	0.2981	0.033*	
H1B	0.0784	0.3701	0.3937	0.033*	0.50
H1C	0.0784	0.1299	0.3937	0.033*	0.50
C2	0.2135 (2)	0.2500	0.3993 (2)	0.0163 (6)	
C3	0.2387 (2)	0.2500	0.4946 (2)	0.0165 (6)	
H3A	0.1889	0.2500	0.5412	0.020*	
C4	0.3340 (2)	0.2500	0.5277 (2)	0.0137 (6)	
C5	0.3508 (2)	0.2500	0.6351 (2)	0.0198 (7)	
H5A	0.4197	0.2500	0.6480	0.030*	
H5B	0.3218	0.1299	0.6635	0.030*	0.50
H5C	0.3218	0.3701	0.6635	0.030*	0.50
C6	0.5624 (2)	0.2500	0.4488 (2)	0.0140 (6)	
C7	0.6634 (2)	0.2500	0.4810 (2)	0.0133 (6)	
C8	0.7378 (2)	0.2500	0.4146 (2)	0.0156 (6)	
H8	0.7246	0.2500	0.3475	0.019*	
C9	0.8314 (2)	0.2500	0.4473 (2)	0.0165 (6)	
H9	0.8828	0.2500	0.4025	0.020*	
C10	0.7787 (2)	0.2500	0.6080 (2)	0.0166 (6)	
H10	0.7941	0.2500	0.6746	0.020*	
C11	0.6845 (2)	0.2500	0.5800 (2)	0.0153 (6)	
H11	0.6347	0.2500	0.6265	0.018*	

### Atomic displacement parameters (Å^2^)

**Table:**

	*U*^11^	*U*^22^	*U*^33^	*U*^12^	*U*^13^	*U*^23^
V1	0.0111 (3)	0.0163 (3)	0.0087 (3)	0.000	0.00041 (18)	0.000
O1	0.0180 (8)	0.0203 (8)	0.0192 (8)	0.0007 (6)	−0.0002 (6)	−0.0048 (7)
O2	0.0131 (11)	0.0351 (14)	0.0129 (11)	0.000	−0.0001 (9)	0.000
O3	0.0127 (11)	0.0333 (14)	0.0108 (11)	0.000	−0.0004 (8)	0.000
O1W	0.0188 (12)	0.0193 (12)	0.0201 (12)	0.000	0.0066 (10)	0.000
N1	0.0109 (12)	0.0183 (13)	0.0121 (13)	0.000	−0.0012 (9)	0.000
N2	0.0121 (12)	0.0171 (12)	0.0124 (12)	0.000	−0.0019 (10)	0.000
N3	0.0126 (12)	0.0192 (13)	0.0146 (13)	0.000	−0.0021 (10)	0.000
C1	0.0122 (15)	0.035 (2)	0.0187 (16)	0.000	−0.0006 (13)	0.000
C2	0.0128 (14)	0.0195 (15)	0.0165 (16)	0.000	0.0011 (12)	0.000
C3	0.0144 (15)	0.0194 (15)	0.0158 (15)	0.000	0.0010 (12)	0.000
C4	0.0154 (15)	0.0141 (14)	0.0115 (14)	0.000	0.0005 (11)	0.000
C5	0.0178 (15)	0.0314 (19)	0.0102 (15)	0.000	0.0025 (12)	0.000
C6	0.0143 (14)	0.0150 (14)	0.0126 (14)	0.000	−0.0013 (12)	0.000
C7	0.0140 (14)	0.0131 (14)	0.0128 (14)	0.000	−0.0016 (11)	0.000
C8	0.0170 (15)	0.0180 (15)	0.0117 (14)	0.000	−0.0009 (12)	0.000
C9	0.0154 (15)	0.0195 (15)	0.0145 (15)	0.000	0.0010 (12)	0.000
C10	0.0185 (15)	0.0191 (15)	0.0123 (15)	0.000	−0.0015 (12)	0.000
C11	0.0156 (15)	0.0181 (15)	0.0122 (14)	0.000	0.0021 (12)	0.000

### Geometric parameters (Å, °)

**Table:**

V1—O1	1.6323 (17)	C1—H1C	0.9800
V1—O1^i^	1.6323 (17)	C2—C3	1.369 (5)
V1—O2	1.938 (2)	C3—C4	1.411 (4)
V1—O3	1.962 (2)	C3—H3A	0.9500
V1—N1	2.115 (3)	C4—C5	1.510 (4)
O2—C2	1.295 (4)	C5—H5A	0.9800
O3—C6	1.310 (4)	C5—H5B	0.9800
O1W—H1W	0.838 (10)	C5—H5C	0.9800
N1—C4	1.325 (4)	C6—C7	1.481 (4)
N1—N2	1.404 (4)	C7—C8	1.391 (4)
N2—C6	1.286 (4)	C7—C11	1.407 (4)
N3—C9	1.340 (4)	C8—C9	1.385 (4)
N3—C10	1.349 (4)	C8—H8	0.9500
N3—H3	0.861 (10)	C9—H9	0.9500
C1—C2	1.507 (4)	C10—C11	1.373 (5)
C1—H1A	0.9800	C10—H10	0.9500
C1—H1B	0.9800	C11—H11	0.9500
			
O1—V1—O1^i^	109.11 (13)	C2—C3—H3A	118.1
O1—V1—O2	96.61 (7)	C4—C3—H3A	118.1
O1^i^—V1—O2	96.61 (7)	N1—C4—C3	121.8 (3)
O1—V1—O3	96.25 (7)	N1—C4—C5	120.3 (3)
O1^i^—V1—O3	96.25 (7)	C3—C4—C5	117.9 (3)
O2—V1—O3	157.73 (10)	C4—C5—H5A	109.5
O1—V1—N1	125.34 (6)	C4—C5—H5B	109.5
O1^i^—V1—N1	125.34 (6)	H5A—C5—H5B	109.5
O2—V1—N1	82.75 (10)	C4—C5—H5C	109.5
O3—V1—N1	74.98 (10)	H5A—C5—H5C	109.5
C2—O2—V1	136.3 (2)	H5B—C5—H5C	109.5
C6—O3—V1	116.6 (2)	N2—C6—O3	124.5 (3)
C4—N1—N2	113.6 (3)	N2—C6—C7	118.0 (3)
C4—N1—V1	130.9 (2)	O3—C6—C7	117.5 (3)
N2—N1—V1	115.46 (19)	C8—C7—C11	119.4 (3)
C6—N2—N1	108.4 (3)	C8—C7—C6	120.9 (3)
C9—N3—C10	122.0 (3)	C11—C7—C6	119.7 (3)
C9—N3—H3	112 (4)	C9—C8—C7	119.3 (3)
C10—N3—H3	126 (4)	C9—C8—H8	120.3
C2—C1—H1A	109.5	C7—C8—H8	120.3
C2—C1—H1B	109.5	N3—C9—C8	120.0 (3)
H1A—C1—H1B	109.5	N3—C9—H9	120.0
C2—C1—H1C	109.5	C8—C9—H9	120.0
H1A—C1—H1C	109.5	N3—C10—C11	120.7 (3)
H1B—C1—H1C	109.5	N3—C10—H10	119.7
O2—C2—C3	124.3 (3)	C11—C10—H10	119.7
O2—C2—C1	114.3 (3)	C10—C11—C7	118.6 (3)
C3—C2—C1	121.4 (3)	C10—C11—H11	120.7
C2—C3—C4	123.9 (3)	C7—C11—H11	120.7
			
O1—V1—O2—C2	124.90 (6)	N2—N1—C4—C3	180.0
O1^i^—V1—O2—C2	−124.90 (6)	V1—N1—C4—C3	0.0
O3—V1—O2—C2	0.0	N2—N1—C4—C5	0.0
N1—V1—O2—C2	0.0	V1—N1—C4—C5	180.0
O1—V1—O3—C6	−124.96 (6)	C2—C3—C4—N1	0.0
O1^i^—V1—O3—C6	124.96 (6)	C2—C3—C4—C5	180.0
O2—V1—O3—C6	0.0	N1—N2—C6—O3	0.0
N1—V1—O3—C6	0.0	N1—N2—C6—C7	180.0
O1—V1—N1—C4	−92.98 (9)	V1—O3—C6—N2	0.0
O1^i^—V1—N1—C4	92.98 (9)	V1—O3—C6—C7	180.0
O2—V1—N1—C4	0.0	N2—C6—C7—C8	180.0
O3—V1—N1—C4	180.0	O3—C6—C7—C8	0.0
O1—V1—N1—N2	87.02 (9)	N2—C6—C7—C11	0.0
O1^i^—V1—N1—N2	−87.02 (9)	O3—C6—C7—C11	180.0
O2—V1—N1—N2	180.0	C11—C7—C8—C9	0.0
O3—V1—N1—N2	0.0	C6—C7—C8—C9	180.0
C4—N1—N2—C6	180.0	C10—N3—C9—C8	0.0
V1—N1—N2—C6	0.0	C7—C8—C9—N3	0.0
V1—O2—C2—C3	0.0	C9—N3—C10—C11	0.0
V1—O2—C2—C1	180.0	N3—C10—C11—C7	0.0
O2—C2—C3—C4	0.0	C8—C7—C11—C10	0.0
C1—C2—C3—C4	180.0	C6—C7—C11—C10	180.0

Symmetry codes: (i) *x*, −*y*+1/2, *z*.

### Hydrogen-bond geometry (Å, °)

**Table:**

*D*—H···*A*	*D*—H	H···*A*	*D*···*A*	*D*—H···*A*
O1W—H1w···O1	0.84 (1)	1.91 (1)	2.732 (2)	168 (3)
N3—H3···O1w^ii^	0.86 (1)	1.87 (3)	2.683 (4)	158 (6)

Symmetry codes: (ii) −*x*+3/2, −*y*, *z*+1/2.
